# PW01-022 – Dissociation between CRP and SAA in FMF

**DOI:** 10.1186/1546-0096-11-S1-A75

**Published:** 2013-11-08

**Authors:** K Stankovic Stojanovic, V Hentgen, S Georgin-Lavialle, S Fellahi, I Jeru, S Amselem, J-P Bastard, G Grateau

**Affiliations:** 1 Internal Medicine, APHP, Hôpital Tenon, Paris, France; 2 Pediatrics, Hopital de Versailles, Le Chesnay, France; 3 Biochemistry Laboratory, APHP, Hôpital Tenon, Paris, France; 4 Genetic Laboratory, APHP, Hopital Trousseau, Paris, France

## Introduction

An Israeli study previously showed that dissociation between normal C-reactive protein (CRP) and elevated serum amyloid A (SAA) could be observed in Familial Mediterranean fever (FMF). Considering that elevated SAA is predictive for AA amyloidosis, this study suggested that SAA could be a better tool in the diagnosis and therapeutic management of FMF.

## Objectives

To analyze the dissociation between CRP and SAA in a large cohort of FMF adults and children in France.

## Methods

CRP and SAA were systematically measured during the follow-up of consecutive attack-free FMF outpatients seen in a pediatric and an adult French reference center. Dissociations between CRP and SAA were defined by normal CRP (<5mg/L) and elevated SAA (group A), or elevated CRP and normal SAA (<10mg/L) (group B). Demographic data, genotype, clinical characteristics of FMF, and treatment were analyzed.

## Results

274 samples from 219 patients were analysed. The cohort had a median age of 24 years old [interquartile 15-35], 54% were female. Ethnic origins were: 60% non-ashkenasi Jews, 1% ashkenasi Jews, 4% mixed, 9.5% Arabs, 5% Armenians, 5% Turks, 3% Lebanese or Syrians, 1% Italians, 1% Portuguese or Spanish, 1% Caucasian. *MEFV* genotype was known in 181 patients (83%): 63.5% had 2 non-ambiguous mutations, 24% were simple heterozygous, 7% were compound heterozygous with one non-ambiguous mutation and one polymorphism, 5.5% had no mutation. Six patients had amyloidosis. 181 patients (83%) were treated with colchicine, 3 patients with interleukin-1 inhibitor. Elevated SAA (median=16.5mg/L [13;31] while normal CRP was found in 21 samples (13% samples of with normal CRP). Elevated CRP (median=9mg/L [7;11]) while normal SAA was found in 38 samples (22% samples of normal SAA). Age was significantly higher in group B comparing to group A or the group with no dissociation (33 years old versus 21 and 23 respectively, p=0.004). Colchicine dosage was significantly higher in group B comparing to the group with no dissociation (1.05mg/day versus 1.34, p=0.04). No statistical difference was found concerning genotype or Ethnic origin. Dissociation with high SAA and normal CRP was found in some patients with amyloidosis but the difference was not statistically different (p=0.08). Finally, for values of CRP above 30mg/L (30-63mg/L), corresponding SAA values were 1.5 to 6 times higher (53-683).

## Conclusion

Dissociation between SAA and CRP was not frequent in our study. Genotype and ethnic origin were not predictive for this dissociation.

## Disclosure of interest

None declared

